# Supplementary material for: Analysis of PPARγ Signaling Activity in Psoriasis
Source: Int J Mol Sci. 2021 Aug 10;22(16):8603. doi: 10.3390/ijms22168603 (PMC8395241; doi:10.3390/ijms22168603)
Supplement: Supplementary file 1 [file ijms-22-08603-s001.zip › Supplemental materials_Analysis of PPARg signaling activity in psoriasis/Pathway models/Models images and html files/PPARG inhibits phenotypes assotiated with psoriasis/132329.html]

injury


# Disease injury

|  |  |
| --- | --- |
| URN | urn:agi-meshdis:Injuries |
| Total Entities | 55 |
| Connectivity | 17688 |
| Name | injury |

---

|  |  |
| --- | --- |
| ChildConcepts | bites and stings |
|  | contusion |
|  | neurological damage |
|  | rupture |
|  | perforation |
|  | abdominal trauma |
|  | abrasion |
|  | accidental injury |
|  | cutaneous injury |
|  | vascular injury |
|  | avulsion |
|  | war trauma |
|  | erosion |
|  | musculoskeletal injury |
|  | breast trauma |
|  | respiratory tract injury |
|  | burn |
|  | chemical injury |
|  | cardiac injury |
|  | contrecoup injury |
|  | crush injury |
|  | thoracic trauma |
|  | foreign body |
|  | urogenital injury |
|  | head and neck injury |
|  | barotrauma |
|  | immune injury |
|  | immune-mediated injury |
|  | radiation damage |
|  | limb injury |
|  | organ damage |
|  | pelvic trauma |
|  | psychological trauma |
|  | seat belt injury |
|  | thermal injury |
|  | traumatic hematoma |
|  | reperfusion injury |
|  | self-injurious behavior |
|  | electrical injury |
|  | amputation stump |
|  | soft tissue injury |
|  | microtrauma |
|  | traumatic amputation |
|  | sports injury |
|  | birth trauma |
|  | traumatic shock |
|  | drowning |
|  | jaw injury |
|  | multiple trauma |
|  | sprains and strains |
|  | tooth injury |
|  | back injury |
|  | preconception injury |
|  | prenatal injury |
|  | surgical trauma |

---

|  |  |
| --- | --- |
| Pathway | PPARG inhibits phenotypes assotiated with psoriasis |
|  | Neighbors of injury |

---

|  |  |
| --- | --- |
| MedScan ID | 9023020 |

---

|  |  |
| --- | --- |
| Alias | traumatic injuries |
|  | traumatic or non-traumatic injuries |
|  | traumatic lesion |
|  | traumatic injury |
|  | injuries |
|  | traumatic or non-traumatic injury |
|  | traumatic lesions |
|  | injury |

---

|  |  |
| --- | --- |
| MeSH ID | M0023020 |

---

|  |  |
| --- | --- |
| MeSH Heading | Injuries |

---
